# Research on a Miniaturized Digital Servo System for Passive Hydrogen Masers

**DOI:** 10.3390/s26072279

**Published:** 2026-04-07

**Authors:** Siyuan Guo, Meng Cao, Pengfei Chen, Tao Shuai, Wangwang Hu, Yuxian Pei

**Affiliations:** 1 School of Materials Science and Engineering, Shanghai University, Shanghai 200444, China; guosiyuan@shu.edu.cn (S.G.); caomeng@shu.edu.cn (M.C.); 2 Shanghai Astronomical Observatory, Chinese Academy of Sciences, Shanghai 200030, China; shuait@shao.ac.cn (T.S.); huwangwang@shao.ac.cn (W.H.); celline@shao.ac.cn (Y.P.)

**Keywords:** passive hydrogen maser, software-defined radio, digital servo, oversampling

## Abstract

**Highlights:**

**What are the main findings?**

- A digital servo architecture for a passive hydrogen maser based on SDR and the FPGA was developed, using the AD9364 transceiver to integrate microwave interrogation signal generation, microwave receiver down-conversion, and analog-to-digital conversion, and stable closed-loop locking of the atomic transition spectrum was achieved.
- By combining dual-frequency time-division probing with outlier rejection and averaging decimation, the atomic and cavity discriminator signals were separated effectively, enabling frequency stability at $1.46\times 10^{-12}$ at a 1 s averaging time while substantially reducing the power consumption and size of the servo electronics.

**What are the implications of the main findings?**

- The results demonstrate that passive hydrogen maser servo electronics can be shifted from conventional discrete analog implementations to a highly integrated digital architecture, providing a feasible route toward compact, low-power, and reconfigurable spaceborne frequency reference systems.
- The proposed architecture confirms the potential of SDR technology for precision time–frequency measurement and atomic clock servo control and provides an electronics foundation for the future engineering and miniaturization of spaceborne passive hydrogen masers.

**Abstract:**

High-precision time and frequency references are essential for satellite navigation, deep-space exploration, and space science missions. To address the large size, high power consumption, and limited integration of conventional Passive Hydrogen Maser (PHM) servo electronics based on discrete analog chains, this paper proposes a miniaturized digital servo architecture for PHMs based on software-defined radio (SDR) and a field-programmable gate array (FPGA). The AD9364 is used as an integrated RF front end for microwave interrogation signal generation, receiver down-conversion, and analog-to-digital conversion (ADC), while digital demodulation, discriminator construction, and closed-loop control are implemented in the FPGA. A dual-frequency interrogation and time-division multiplexing scheme is introduced to separate the atomic and cavity responses, and an oversampling-based processing method combining outlier rejection and averaging decimation is adopted to improve the observation accuracy and noise immunity of weak error signals. Experimental results demonstrate stable closed-loop locking of the atomic transition spectrum, achieving a frequency stability of 1.46 × 10^−12^ at 1 s, while significantly improving the compactness and integration level of the servo electronics.

## 1. Introduction

The Passive Hydrogen Maser (PHM), owing to its excellent short- and medium-term frequency stability, has become one of the most important candidates for high-performance spaceborne frequency references [1]. In applications such as global navigation satellite systems, deep-space exploration, and space very long baseline interferometry (VLBI), high-precision time and frequency references are directly related to ranging accuracy, phase coherence, and long-term autonomous operation capability. Compared with conventional spaceborne frequency standards such as rubidium atomic clocks, the PHM exhibits clear advantages in terms of frequency drift and short- to medium-term stability. Consequently, PHMs have been deployed in systems such as Galileo and BeiDou-3 [2,3,4], and are being further extended to advanced mission scenarios including lunar-orbit VLBI, high-precision deep-space measurement, and gravitational redshift tests [5,6,7,8]. As space missions continue to evolve toward higher precision, greater autonomy, and longer lifetime, the PHM remains a promising candidate for future high-performance spaceborne timing payloads [9].

For microsatellites and emerging low-cost space platforms, limited payload resources make the miniaturization, low power consumption, and high integration of atomic clock systems increasingly important. In recent years, extensive efforts have been devoted to the miniaturization of the PHM physics package. Representative approaches include reducing the volume of the microwave cavity by using high-permittivity, low-loss materials [10,11], advancing the engineering implementation of compact space passive hydrogen masers [12,13], and optimizing the vacuum unit structure and magnetic shielding system to reduce overall size and resource consumption [14,15,16].

However, compared with the continuous optimization of the physics package, servo electronics still generally rely on discrete analog architectures inherited from ground-based systems. Conventional servo chains are typically composed of cascaded modules such as microwave sources, analog mixers, filters, detectors, and control circuits, resulting in long signal paths [17,18], large component counts, complex coupling relationships, and strong sensitivity to environmental variations. This implementation not only increases system volume, power consumption, and debugging complexity, but also limits further engineering integration. As a result, it is becoming increasingly difficult to satisfy the demand for highly integrated electronics in next-generation space platforms.

SDR technology, characterized by highly integrated radio-frequency front ends and flexible reconfigurable digital signal processing capability, has gradually expanded from conventional communication systems to precision measurement and frequency control applications [19,20,21,22]. Compared with traditional implementations relying on multistage analog frequency conversion, narrowband filtering, and independent detection circuits, an SDR-based architecture can unify frequency translation, bandwidth selection, digital demodulation, and servo control algorithms on a programmable hardware platform. This feature provides a new technical route for reconstructing PHM servo electronics in a compact and digital form. For PHM systems, the processes of microwave interrogation signal generation, response acquisition, discriminator construction, and feedback regulation can all be reformulated within a unified SDR framework. Recent studies on digital control and automatic tuning of hydrogen masers further support the feasibility of this direction [23,24,25,26].

To address these issues, this paper proposes a PHM digital servo architecture based on the AD9364 agile RF transceiver and the Xilinx Kintex-7 XC7K325T FPGA. Firstly, an integrated SDR platform for PHM servo electronics is developed, in which microwave interrogation signal generation, microwave receiver down-conversion, digital demodulation, and closed-loop control are incorporated into a unified hardware architecture. Secondly, a dual-frequency interrogation and time-division multiplexing mechanism is adopted to enable the separated extraction of discriminator outputs corresponding to the atomic response and the microwave cavity response. Thirdly, to mitigate the susceptibility of weak error signals to noise interference, an oversampling-based processing method combining outlier rejection and averaging decimation is introduced to improve the anti-interference capability and effective resolution of the error signal. Experimental results demonstrate that the proposed system achieves stable closed-loop locking of the atomic transition spectrum and verifies the feasibility of the proposed architecture in terms of both frequency stability and SWaP performance. It should be emphasized that this work focuses on the digital implementation of the servo electronics, and the experimental study is conducted on an existing passive hydrogen maser system available in our group. In this sense, the digitalization considered here pertains to the servo electronics rather than the entire PHM system. Its eventual limitations are set by the indispensable physical package, the necessary analog/RF interfaces, and the performance of the ADC, DAC, and other front-end hardware.

## 2. Servo Principle and Overall Architecture of PHM

### 2.1. Timekeeping Principle of Hydrogen Maser

**Figure 1:**
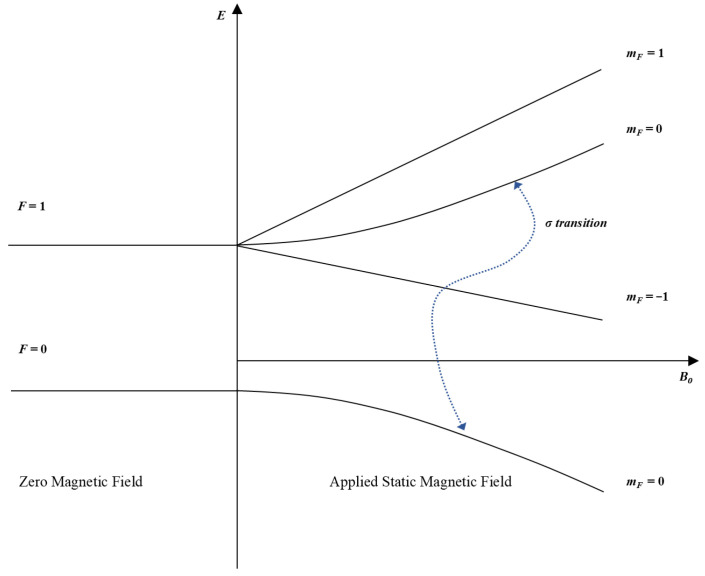
Hyperfine energy-level structure of hydrogen and clock transition used in PHM.

The physical basis of the PHM lies in the electromagnetic interaction between the hyperfine levels of the hydrogen ground state. Molecular hydrogen is first dissociated into atomic hydrogen by radio-frequency discharge. The resulting atoms are then focused by the gradient magnetic field of a state selection magnet, and those in the $F=1,\;m_{F}=0$ and $F=1,\;m_{F}=1$ states are injected into a Teflon-coated quartz storage bulb. Within this enclosed environment, the atoms interact with the microwave field sustained in the resonant cavity, where stimulated emission occurs. Under a weak static magnetic field, the F=1 hyperfine level is further split by the Zeeman effect into three magnetic sublevels corresponding to $m_{F}=-1$, 0, and +1, whereas the F=0 level remains unsplit. According to the Breit–Rabi formula, under weak-field conditions, the clock transition of the hydrogen ground-state hyperfine structure can be expressed as follows:

(1) $$F=1,\;m_{F}=0↔F=0,\;m_{F}=0,$$

and the corresponding transition frequency is given by

(2) $$\nu_{0}=\frac{\Delta E}{h}\approx 1.42040575177\;\mathrm{GHz},$$

where ΔE denotes the energy difference between the two corresponding energy levels, and h is Planck’s constant. Because this transition is insensitive to first-order external magnetic-field perturbations and is fundamentally determined by the intrinsic internal interactions of the hydrogen atom, it exhibits extremely high inherent frequency stability. Therefore, it is adopted as the frequency reference of the PHM. The corresponding physical transition process and hyperfine energy-level structure are illustrated in Figure 1.

**Figure 2:**
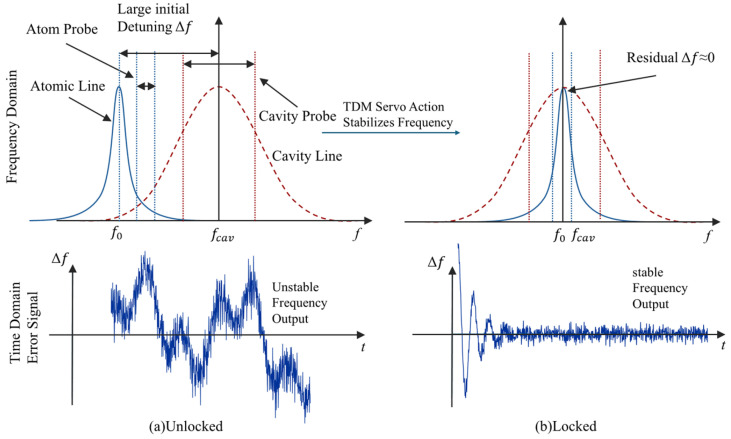
The principle of dual-frequency interrogation and locking. Symmetric interrogation frequencies are applied on both sides of the resonance center, and the response difference is used to generate the discriminator error signal. The lower panels compare the unlocked and locked states in the time domain.

For the PHM, the interrogation microwave is provided by an external signal source. The system does not rely on self-oscillation inside the microwave cavity; instead, it generates an error signal by detecting the variation in the atomic transition response to the applied interrogation signal and then uses this error signal to correct the local frequency source. The atomic transition spectrum of the PHM exhibits a Lorentzian line shape, which can be expressed as

(3) $$g\left(v\right)=\frac{2T_{2}}{1+4\pi^{2}T_{2}^{2}(v-v_{0})},$$

where $T_{2}$ is the transverse relaxation time, ν is the interrogation microwave frequency, and $\nu_{0}$ is the atomic resonance center frequency. As illustrated in Figure 2, the proposed frequency discrimination mechanism is analogous to the slope detection principle. In this scheme, two symmetric interrogation frequencies are applied on the opposite slopes of the atomic resonance line through the external microwave excitation. When a small frequency offset occurs, the response amplitudes at these two frequencies become unequal, thereby providing the basis for discriminator construction and subsequent servo locking.

To further characterize the conversion capability from frequency deviation to error signal, the discriminator sensitivity can be defined as

(4) $$K_{d}=\frac{dV_{e}}{df},$$

where $V_{e}$ is the DC error signal at the detector output and f is the microwave excitation frequency. A larger $K_{d}$ indicates that a larger error signal amplitude can be obtained under the same frequency perturbation, which is beneficial for improving the sensitivity and frequency resolution of the servo loop.

### 2.2. Overall Structure of Dual-Frequency Digital Servo

In the conventional single-frequency interrogation scheme, the response of the atomic hyperfine transition and that of the microwave cavity resonance strongly overlap in the frequency domain, making it difficult to separate their respective discriminator signals effectively. As a result, the atomic and cavity parameters become strongly coupled, which is unfavorable for independent loop adjustment. To address this issue, a digital servo scheme combining dual-frequency interrogation with time-division multiplexing is adopted in this work, as shown in Figure 2. During the dual closed-loop locking process of the atomic transition and the microwave cavity, two pairs of interrogation frequency points, symmetrically distributed around the corresponding resonance centers, are applied in a time-shared manner. By exploiting the local frequency discrimination characteristics of the atomic spectral line and the microwave cavity response curve on the opposite sides of their respective resonance centers, the stimulated response amplitudes at the two symmetric frequency points are compared at the receiver to construct a discriminator signal proportional to the frequency deviation. The corresponding error signal can be written as

(5) $$e(n)=k\left[A_{+}(n)-A_{-}(n)\right],$$

where $A_{+}(n)$ and $A_{-}(n)$ denote the stimulated response amplitudes at the two symmetric interrogation points on either side of the resonance center, respectively, and k is the overall gain coefficient of the amplification, detection, and synchronous demodulation chain. When the two response amplitudes are equal, the error signal becomes zero, indicating that the corresponding interrogation branch is locked to its target resonance center. According to the active interrogation window, the resulting discriminator outputs are then directed to different feedback paths for atomic frequency correction and cavity frequency tuning.

**Figure 3:**
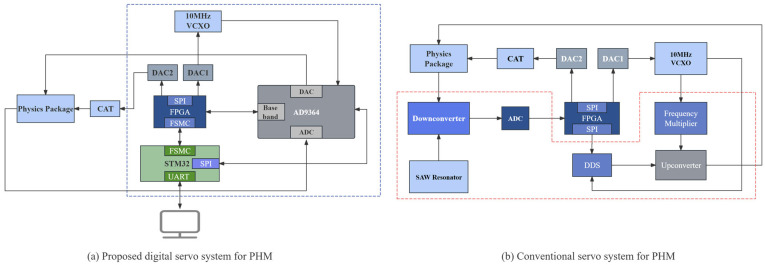
The overall architecture of the proposed SDR-based digital servo system and comparison with a conventional servo structure: (**a**) the proposed digital servo system for the PHM; (**b**) a conventional servo system for the PHM. The red dashed box marks the portion of the conventional architecture replaced by the AD9364-based integrated SDR front end.

Based on the above principle, the overall architecture of the digital servo system is constructed as shown in Figure 3a. The system mainly consists of three parts, the interrogation signal generation and transmission chain, the reception and digital demodulation chain, and the servo control chain, which together realize the closed-loop process of “excitation–response–error control”. To further clarify the architectural improvement in the proposed system, Figure 3 compares the present SDR-based implementation with a conventional servo structure. In the conventional scheme, the interrogation and reception paths are realized by multiple discrete functional modules. On the interrogation side, the 10 MHz VCXO serves as the reference frequency source, and frequency synthesis and frequency conversion are implemented by a separate Direct Digital Synthesizer (DDS) chip, a frequency multiplier, and an upconverter. On the reception side, the response signal from the physics package first passes through a discrete down-conversion stage consisting of a mixer, and is then digitized by a standalone ADC before being delivered to the FPGA for subsequent digital processing and servo computation. Therefore, although FPGA-based digital control can already be introduced into such architectures, the RF front end remains largely discrete.

As highlighted by the red dashed box in Figure 3b, the corresponding discrete modules in the conventional architecture are replaced in the proposed design by the AD9364-based integrated SDR front end. In the proposed architecture, these previously separated RF and data conversion functions are largely integrated into the AD9364, which serves as an integrated SDR front end for both interrogation signal transmission and response signal reception. The interrogation signal is generated and transmitted through the AD9364, while the response signal produced by the physical package is received, down-converted, and digitized by the AD9364 before being output to the FPGA in the form of complex baseband I/Q data. The FPGA then sequentially performs digital down-conversion, multistage decimation filtering, envelope extraction, synchronous demodulation, error construction, and digital servo computation. Finally, the control signal of the atomic servo branch is applied to the frequency control terminal of the VCXO, so that the VCXO is locked to the hydrogen atomic transition signal. Meanwhile, the control quantity of the cavity servo branch is applied to the tuning terminal of the microwave-cavity varactor diode, so that the microwave cavity is tuned and locked with respect to the VCXO output signal. Compared with the conventional discrete architecture, the proposed system not only preserves the flexibility of digital servo control, but also shortens the signal chain, reduces the number of discrete devices and interconnections, and improves the overall integration level of the passive hydrogen maser servo electronics.

**Figure 4:**
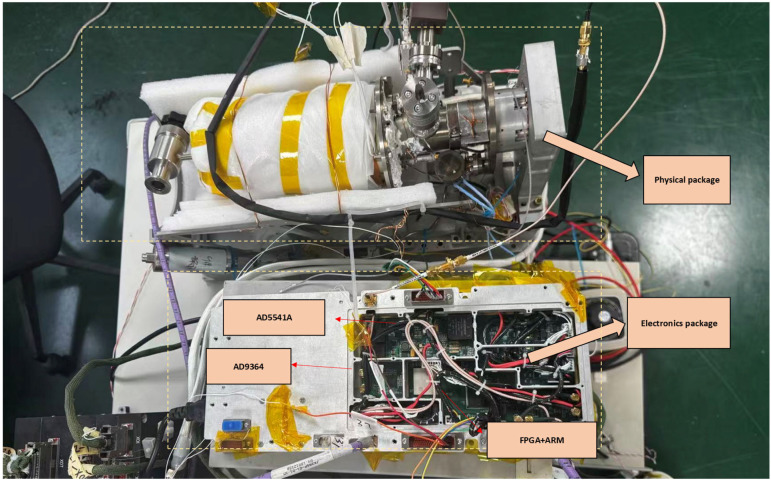
Experimental SDR-based PHM system and its main hardware modules.

Based on the system-level architecture described above, the corresponding hardware implementation is shown in Figure 4. As shown in Figure 4, the hardware implementation adopts the Xilinx Kintex-7 XC7K325T FPGA as the real-time digital processing core, responsible for data acquisition, decimation filtering, synchronous demodulation, and digital servo computations. An AD9364 is used as an integrated RF front end to perform interrogation-signal transmission, receiver down-conversion, and analog-to-digital/digital-to-analog conversion. The main controller, an STM32H743, communicates with the FPGA through the Flexible Static Memory Controller (FSMC) bus, and configures and monitors the AD9364 through a Serial Peripheral Interface (SPI) bus. The VCXO and the microwave cavity tuning branches are driven by dedicated 16-bit AD5541A digital-to-analog converters, ensuring high servo resolution.

## 3. Software-Defined Radio Servo System Design

### 3.1. Interrogation Signal Generation and Transmission Chain

The baseband interrogation excitation signal is generated in the digital domain. A complex baseband sequence is synthesized in the FPGA using a numerically controlled oscillator (NCO)-based digital frequency synthesis module. The instantaneous complex baseband signal can be expressed as a discrete frequency-hopping signal controlled by a state machine:

(6) $$s(t)=A(t)e^{j2\pi \left(f_{bb}+\Delta f(t)\right)t},$$

where A(t) denotes the excitation amplitude controlled by the timing sequence, $f_{bb}$ is the center reference frequency, and Δf is the instantaneous frequency offset. Within a complete interrogation cycle, both the signal amplitude and the frequency offset are switched segment by segment according to the timing schedule:

(7) $$A\left(t\right)=\{\begin{array}{cc} A_{atom}, & 0\leq t\leq T_{atom},\\ A_{cav}, & T_{atom}<t\leq T_{cycle}, \end{array}$$

(8) $$\Delta f\left(t\right)=\{\begin{array}{cc} f_{osc}\cdot (-1{)}^{n}, & 0\leq t\leq T_{atom},\\ f_{cav}\cdot (-1{)}^{n}, & T_{atom}<t\leq T_{cycle}, \end{array}$$

where n=⌊t/T⌋ is the discrete time-step index, T is the frequency switching interval, and $f_{osc}$ and $f_{cav}$ denote the corresponding modulation depths. Considering that the average lifetime of hydrogen atoms in the storage bulb is approximately 1 s, whereas the time constant of the microwave cavity is about 10 μs, frequency switching interval is set to T=40 ms in this work to ensure the continuity of the atomic excitation process while allowing the cavity response to fully settle after each frequency switch. In addition, to compensate for the signal-to-noise-ratio degradation caused by the broader cavity response curve, the power of the cavity frequency interrogation signal is set to be 10 dB higher than that of the atomic interrogation signal. The detailed timing and modulation parameters are listed in Table 1.

**Figure 5:**
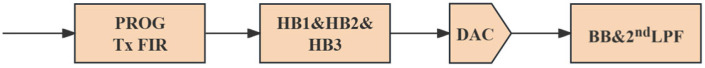
Signal flow of the interrogation signal generation and transmission chain.

Figure 5 illustrates the generation process of the interrogation RF signal. The baseband signal is represented by 12-bit digital data, which is first interpolated by the DUC and then converted into an analog signal by the DAC. The analog signal is subsequently further filtered and up-converted to a higher frequency. Finally, after power adjustment by a programmable attenuator, it is injected into the microwave cavity.

It should be noted that, prior to closed-loop servo operation, an appropriate interrogation power operating point must first be determined through open-loop testing to avoid response nonlinearity and degraded locking performance caused by insufficient excitation or overdrive.

### 3.2. Digitization and Quadrature Demodulation in the Receiver Front End

**Figure 6:**
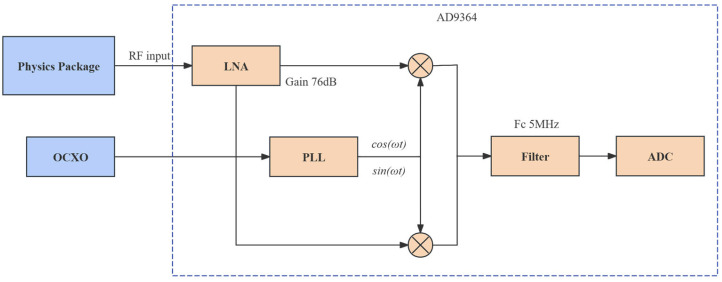
Internal signal processing configuration of the AD9364 receiver front end.

The input RF signal is down-converted and digitized by the AD9364 receiver front end, and the corresponding processing flow is shown in Figure 6. The RF signal at approximately 1.420405 GHz enters the on-chip analog quadrature mixer, where it is frequency-translated under the drive of the quadrature local oscillator synthesized by the internal phase-locked loop, thereby down-converting the target signal to the low–intermediate-frequency (low-IF) region. To suppress spectral aliasing introduced by sampling, an on-chip third-order Butterworth analog baseband low-pass filter is configured ahead of the ADC, with the cutoff frequency set to 5 MHz. After analog-domain frequency translation and bandwidth limitation, the signal is fed into the on-chip ADC and digitized at a sampling rate of 300 MSPS.

For the designed PHM servo system, the center frequency of the down-converted low-IF signal is set to 405 kHz. By combining the analog in-phase and quadrature channels, the resulting complex low-IF signal can be expressed as

(9) $$z(t)=I_{a}(t)+jQ_{a}(t)=\frac{A+M(t)}{2}e^{j(2\pi f_{IF}t+\phi_{0})},$$

where A denotes the DC-equivalent amplitude, M(t) is the low-frequency envelope component introduced by interrogation modulation, and $\phi_{0}$ is the initial phase. After sampling, the corresponding discrete complex sequence is obtained as

(10) $$z[n]=I[n]+jQ[n]=\frac{A+M[n]}{2}e^{j\left(\frac{2\pi f_{IF}n}{f_{s0}}+\phi_{0}\right)}.$$

To obtain a zero-IF complex baseband signal and reduce the influence of residual frequency offset on subsequent envelope extraction and discrimination, an NCO is introduced in the FPGA to perform digital frequency translation, thereby generating a digital complex local oscillator:

(11) $$w[n]=e^{-j2\pi f_{NCO}n/f_{s0}},$$

where $f_{NCO}=405\;\mathrm{kHz}$. The residual frequency offset is defined as

(12) $$\Delta f=f_{IF}-f_{NCO}.$$

Accordingly, the digital down-conversion process can be written as

(13) $$z_{b}[n]=z[n]\cdot w[n]=\frac{A+M[n]}{2}e^{j\left(2\pi \Delta fn/f_{s0}+\phi_{1}\right)},$$

where $\phi_{1}$ denotes the equivalent phase term introduced by the mixing and filtering processes. By defining $I_{b}[n]=R\{z_{b}[n]\}$ and $Q_{b}[n]=I\{z_{b}[n]\}$, the complex baseband quadrature components can be obtained. When Δf=0, the carrier rotation term is eliminated, and $I_{b}[n]$ and $Q_{b}[n]$ mainly reflect the low-frequency envelope information, thereby providing a stable input for subsequent error signal extraction.

### 3.3. Oversampling and Decimation Filtering Design for Error Signal Processing

The ultimate frequency stability of the PHM is directly constrained by the system’s ability to resolve the atomic transition center frequency deviation. The detection response output from the physics package, after analog down-conversion, yields a low-frequency envelope carrying the frequency deviation information of the atoms and the microwave cavity. This error signal has a weak amplitude and is highly susceptible to contamination from quantization noise, thermal noise, and front-end electromagnetic interference. Relying directly on the raw analog-to-digital converter (ADC) sampling to construct the servo discriminant cannot meet the requirements for high-precision locking.

The ADC reference voltage in the AD9364 adopted by the system is 0.625 V, and the quantization resolution is 12 bits; thus, its theoretical quantization step size is

(14) $$\Delta =\frac{V_{ref}}{2^{N}}=\frac{0.625}{2^{12}}\approx 0.153\;\mathrm{mV},$$

where $V_{ref}$ is the reference voltage and N is the nominal ADC resolution. According to Equation (14), the minimum theoretically resolvable voltage of the system is approximately 0.153 mV. However, in practical measurements, the error signal is usually superimposed with broadband random noise and disturbances from the analog front end. If the noise fluctuation reaches approximately 5 mV, the corresponding quantization jitter in units of least significant bits (LSBs) can be estimated as

(15) $$N_{LSB}=\frac{5\;\mathrm{mV}}{0.153\;\mathrm{mV}}\approx 32.7.$$

This indicates that the lower-order quantization bits will be significantly disturbed, thereby limiting the effective observation resolution of the error signal. For a hydrogen atomic clock servo system that relies on weak discriminator signals to achieve high-precision locking, this loss of resolution will directly reduce the purity of the error signal and further affect the short-term stability of the system.

**Figure 7:**
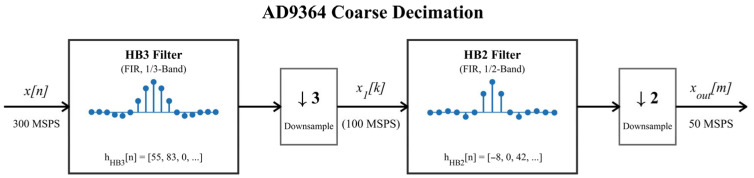
Cascaded HB3/HB2 decimation filtering in the AD9364 digital front end.

To improve the extraction accuracy of weak error signals, the high-speed sampling capability of the AD9364 receiver is exploited to oversample the error signal. As shown in Figure 7, the system sampling rate is set to 300 MSPS, and the sampled data are then decimated by the on-chip HB3 and HB2 filters, reducing the output rate to 50 MSPS. This process not only lowers the data throughput of the subsequent processing stages, but also performs pre-decimation bandwidth limiting, thereby suppressing out-of-band noise and preventing aliasing during down-sampling. The multistage decimation process can be expressed as follows:

(16) $$\{\begin{array}{c} y_{1}[n]=(x*h_{HB3})[n],\\ x_{1}[k]=y_{1}[3k],\\ y_{2}[k]=(x_{1}*h_{HB2})[k],\\ x_{out}[m]=y_{2}[2m], \end{array}$$

where $h_{HB3}[n]$ and $h_{HB2}[n]$ denote the impulse responses of the two decimation filters, respectively, “*” denotes the discrete convolution operation, and $x_{out}[m]$ is the final decimated output sequence.

For an oversampling system, the oversampling ratio (OSR) can be defined as the ratio of the sampling frequency to twice the signal bandwidth, i.e.,

(17) $$OSR=\frac{f_{s}}{2B},$$

where $f_{s}$ is the sampling frequency and B is the effective bandwidth of the target signal. If the quantization noise is assumed to be approximately uniformly distributed over the Nyquist bandwidth, the total quantization noise power can be written as

(18) $$P_{q}=\frac{\Delta^{2}}{12},$$

whereas the in-band quantization noise power falling within the effective signal bandwidth can be approximately expressed as

(19) $$P_{q,in}=\frac{P_{q}}{OSR}.$$

**Figure 8:**
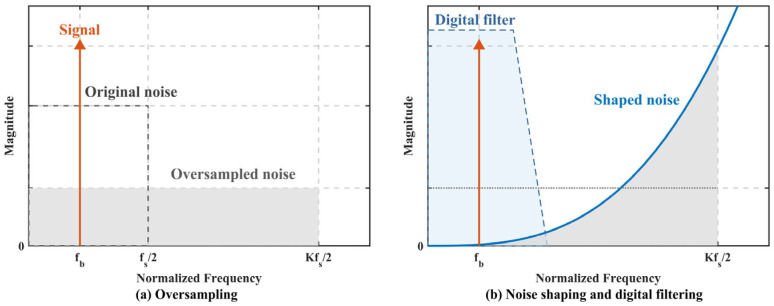
Spectral interpretation of oversampling and quantization noise suppression: (**a**) oversampling; (**b**) ΣΔ noise shaping.

The core discriminant quantity of the servo loop is the ultra-low-frequency error envelope superimposed on the carrier, particularly the characteristic modulation component at 12.5 Hz. Because the target bandwidth is extremely narrow, the redundant data generated by high-speed sampling can be converted into processing gain through subsequent decimation and averaging. Figure 8a illustrates how sampling far above the Nyquist rate spreads the quantization noise over a wider frequency band. Figure 8b shows the noise-shaping mechanism of the ΣΔ ADC, in which high-frequency noise is pushed out of band and the in-band noise floor can be further reduced by digital low-pass filtering.

On this basis, after the AD9364 completes two-stage on-chip decimation, the sampled sequence is delivered to the FPGA for subsequent digital demodulation and envelope extraction. Considering that this sequence may still be affected by transient spikes, abnormal quantization jumps, and local outlier samples, a window-based statistical module using trimmed averaging is further introduced in the FPGA to perform robust decimation of the envelope sequence.

Let the window length be $N=2^{m}$. For the input sequence in the k-th window, after removing the maximum and minimum samples, the trimmed-mean output is given by

(20) $$y\left[k\right]=\frac{1}{N-2}\left(\sum_{n=0}^{N-1}x\left[kN+n\right]-x_{max}\left[k\right]-x_{min}\left[k\right]\right).$$

In practical FPGA implementation, since N≫2, the factor 1/(N−2) can be approximated by 1/N to simplify hardware division. Accordingly, the output is implemented as

(21) $$y\left[k\right]\approx \frac{1}{N}S_{trim}\left[k\right]=S_{trim}\left[k\right]\gg m,$$

where:

(22) $$S_{trim}\left[k\right]=\sum_{n=0}^{N-1}x\left[kN+n\right]-x_{max}\left[k\right]-x_{min}\left[k\right]$$

The relative gain error introduced by replacing 1/(N−2) with 1/N is negligible when N is sufficiently large.

**Figure 9:**
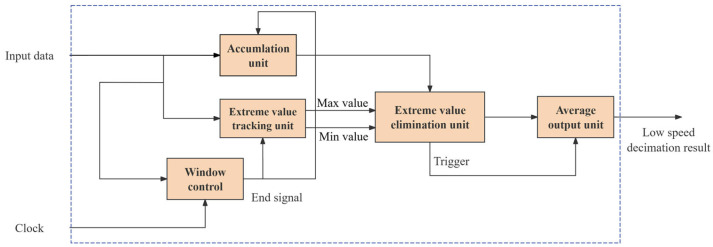
FPGA implementation of proposed outlier rejection and averaging decimation method.

As shown in Figure 9, the proposed algorithm is implemented in the FPGA using a streaming window-based statistical architecture, which mainly consists of window accumulation, extreme-value tracking, window control, extreme-value removal, and averaging output units. The window accumulation unit computes the sum of input samples, while the extreme-value tracking unit records the maximum and minimum values within each window in parallel. The window control unit generates the end-of-window signal, and the extreme-value removal and averaging output unit produces the low-rate decimated result at the end of each window.

Although the trimmed-mean operation is not strictly a linear time-invariant filtering process, its dominant smoothing effect can be approximately represented by an N-point moving-average low-pass filter, whose z-domain transfer function is expressed as

(23) $$H(z)=\frac{1}{N}\sum_{n=0}^{N-1}z^{-n}=\frac{1}{N}\cdot \frac{1-z^{-N}}{1-z^{-1}}.$$

**Figure 10:**
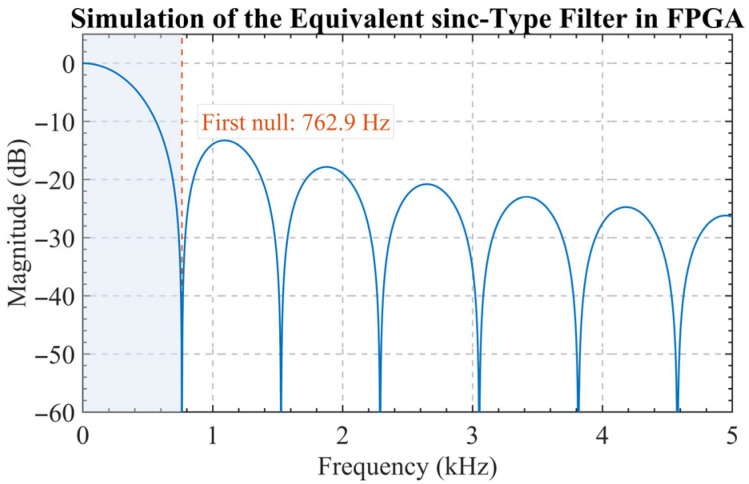
Simulated frequency response of FPGA decimation filter.

Since $z=e^{j\omega }$, the corresponding magnitude response is obtained as follows:

(24) $$|H\left(e^{j\omega }\right)|=\frac{1}{N}|\frac{sin(N\omega /2)}{sin(\omega /2)}|$$

which exhibits a typical sinc-shaped characteristic. It maintains a high gain in the low-frequency region while providing significant attenuation in the high-frequency region, as shown in Figure 10. Therefore, it preserves the low-frequency useful components in the envelope while achieving substantial averaging suppression of broadband random noise. After this processing, the output data rate is reduced from the input sampling rate $f_{s}$ to

(25) $$f_{out}=\frac{f_{s}}{N}.$$

Under the ideal assumption that the in-band noise is dominated by uncorrelated white noise, the noise power after averaging is approximately reduced by a factor of N. The corresponding upper-bound improvement in effective number of bits (ENOB) can be estimated as

(26) $$\Delta ENOB\approx \frac{1}{2}{log}_{2}N.$$

For N=65,536, this idealized estimate corresponds to an ENOB improvement of approximately 8 bits. In practice, however, the actual improvement may be smaller due to correlated noise, residual interference, and non-ideal analog front-end effects.

### 3.4. Error Signal Extraction and Incremental PI Servo Control

Based on the dual-frequency interrogation and discrimination principle described in Section 2, after obtaining the low-rate continuous envelope output sequence following outlier rejection and averaging decimation, the system synchronously extracts and separates the response amplitudes corresponding to different interrogation frequency points according to the predefined modulation timing sequence. On this basis, digital error signals for the atomic frequency control branch and the microwave cavity tuning branch are further constructed, and closed-loop locking is ultimately achieved through a digital controller. The complex baseband signal after digital down-conversion can be expressed as

(27) x[n]=I[n]+jQ[n],

where I[n] and Q[n] denote the in-phase and quadrature components, respectively. To characterize the stimulated response strength at the current interrogation frequency, the envelope amplitude is extracted by magnitude calculation as

(28) $$A\left[n\right]=\sqrt{I^{2}\left[n\right]+Q^{2}\left[n\right]},$$

where A[n] denotes the instantaneous envelope amplitude at the n-th sampling instant. In the FPGA, the low-rate envelope sequence obtained after outlier rejection and averaging decimation is synchronously separated according to the dual-frequency interrogation schedule, yielding the averaged response amplitudes $A_{+}[k]$ and $A_{-}[k]$ at the two symmetric interrogation points around the resonance center. The differential error signal in the k-th control cycle is therefore given by

(29) $$e[k]=A_{+}[k]-A_{-}[k].$$

Owing to the time-division interrogation scheme, the discriminator outputs associated with the atomic and cavity branches are naturally separated in different time slots. Accordingly, the error signal extracted during the atomic interrogation interval is denoted by $e_{atom}[k]$ and fed to the atomic frequency control loop, while the error signal extracted during the cavity interrogation interval is denoted by $e_{cavity}[k]$ and delivered to the cavity tuning loop. This time-domain demultiplexing mechanism ensures effective decoupling of the two servo branches in the digital domain.

An incremental PI controller is adopted for loop regulation. Let u[k] denote the control output in the k-th control cycle. The control increment is defined as

(30) Δu[k]=u[k]−u[k−1],

and its discrete form can be written as

(31) $$\Delta u[k]=K_{p}(e[k]-e[k-1])+K_{i}e[k],$$

where $K_{p}$ and $K_{i}$ are the proportional and integral gains, respectively. The control output is then updated as

(32) u[k]=u[k−1]+Δu[k].

Substituting (31) into (32), the incremental PI control law is obtained as

(33) $$u\left[k\right]=u\left[k-1\right]+K_{p}\left(e\left[k\right]-e\left[k-1\right]\right)+K_{i}e\left[k\right].$$

Within each control update cycle, the low-rate envelope sequence is first synchronously separated according to the dual-frequency interrogation timing to obtain the corresponding averaged response amplitudes $A_{+}[k]$ and $A_{-}[k]$. The differential error signal is then constructed and fed into the corresponding incremental PI controller. During the atomic interrogation interval, the extracted error signal $e_{atom}[k]$ is used to update the control output $u_{atom}[k]$, which is sent to the DAC and applied to the VCXO tuning port. During the cavity interrogation interval, the extracted error signal $e_{cavity}[k]$ is used to update the control output $u_{cavity}[k]$, which is applied to the microwave cavity varactor tuning port. In this way, the atomic and cavity servo branches share the same control law while remaining temporally separated, thereby realizing digitally decoupled closed-loop control.

## 4. Experiment and Analysis

### 4.1. Atomic Spectral-Line Evaluation and Operating Point Determination

Before the closed-loop operation is established, the system first undergoes open-loop initialization. The microwave cavity response is first scanned, and the control voltage corresponding to the peak demodulated amplitude is selected as the initial bias point for the cavity-frequency control loop. With the cavity operating point fixed, the atomic transition spectral line is then scanned, and the region near the spectral-line peak is taken as the initial operating range for establishing the atomic frequency closed loop.

To compare the measured atomic spectral lines under different operating conditions in a concise and engineering-oriented manner, an empirical spectral-line quality metric, denoted by R, is introduced in this study. This metric is not intended as a universal physical parameter of the atomic resonance itself; rather, it is used here as a practical figure of merit to jointly reflect the combined effects of spectral-line amplitude and linewidth during parameter optimization. It is defined as

(34) $$R=10{log}_{10}\left(\frac{(V_{peak}-V_{trough}{)}^{2}}{\Delta f}\right),$$

where $V_{peak}$ and $V_{trough}$ are the maximum and minimum response values of the spectral line, respectively, and Δf is the characteristic linewidth. A larger R indicates a larger spectral-line amplitude and a narrower linewidth, corresponding to better discrimination performance.

In the data processing procedure, the atomic spectral-line data within the scanning range are first extracted, and the scan control code is converted into a relative frequency coordinate, while the sampled output is converted into the corresponding voltage response. The peak value $V_{peak}$ and trough value $V_{trough}$ are then identified, and the amplitude is calculated as

(35) $$A=V_{peak}-V_{trough}.$$

The linewidth is determined from the frequency difference between the left and right intersection points of the spectral curve at

(36) $$V_{t}=V_{trough}+\frac{V_{peak}-V_{trough}}{\sqrt{2}},$$

which is denoted as Δf. Finally, the amplitude and linewidth of each data set are substituted into (34) to calculate the corresponding R value, and the parameter setting that yields the maximum R is selected as the optimum operating point of the atomic spectral line.

**Figure 11:**
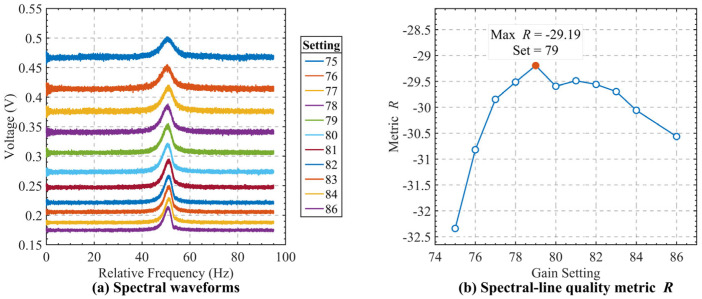
Atomic resonance evaluation under different gain settings: (**a**) resonance responses; (**b**) spectral quality metric R.

Figure 11 shows the measured atomic spectral-line response curves under different external excitation power settings, together with the corresponding values of the spectral-line quality metric R. As shown in Figure 11a, the spectral-line amplitude varies significantly with the excitation power, indicating that the external power level has a direct influence on the observed atomic response. To quantitatively compare the spectral-line quality under different settings, the empirical metric R, which jointly characterizes the spectral-line amplitude and linewidth, is calculated for each case. As shown in Figure 11b, R first increases and then decreases with increasing excitation power, reaching its maximum at an external excitation power of −79 dBm. This result indicates that −79 dBm provides the most favorable balance between response amplitude and spectral-line width among the tested conditions, and is therefore selected as the optimum interrogation power.

### 4.2. Noise Suppression and Equivalent Resolution Improvement in Error Signal

After determining the operating point of the atomic spectral line, a comparative analysis of the error signal before and after digital processing was further carried out to verify the improvement in weak error signal observation accuracy achieved by the proposed outlier rejection and averaging decimation method. All relevant data were acquired using the internal logic analyzer (ILA) embedded in the FPGA. The raw error signal was taken from the envelope output before digital processing, whereas the processed error signal was taken from the low-rate output sequence after outlier rejection and averaging decimation inside the FPGA.

**Figure 12:**
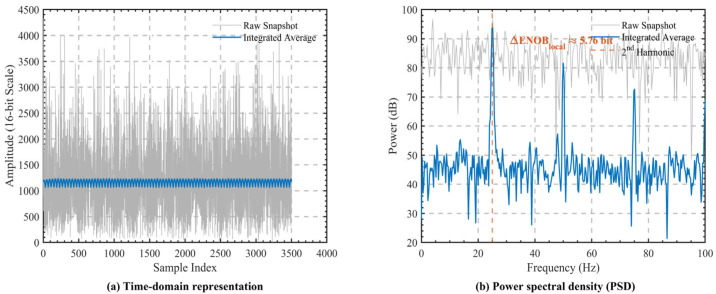
Error signal comparison before and after digital processing: (**a**) time-domain waveform; (**b**) PSD.

Figure 12a shows the time-domain comparison of the error signal before and after digital processing. Before processing, the raw error signal exhibits pronounced random fluctuations, with broadband noise dominating the waveform and the weak low-frequency discriminant information being submerged in the strong noise background. Experimental results show that the peak-to-peak value of the raw error signal is approximately 720 LSB. After applying the proposed outlier rejection and averaging decimation method, the fluctuation amplitude of the output error signal is significantly reduced, and the noise swing is compressed to within approximately 15 LSB. This indicates that, after outlier rejection and averaging decimation, the random noise in the continuous envelope samples is effectively suppressed, and the discernibility of the error signal is markedly improved. To further evaluate the frequency-domain noise suppression capability of the proposed method, the power spectral density (PSD) of the residual error signal under steady-state frequency-locking conditions was analyzed, as shown in Figure 12b. The PSD of the raw error signal exhibits a typical broadband noise-dominated characteristic over the analyzed frequency range, with a relatively high noise floor. After digital processing, the noise floor is significantly reduced across the entire analyzed band, corresponding to an in-band noise power suppression of approximately 34.68 dB. According to the approximate conversion relationship between noise suppression and effective resolution, this result indicates that the proposed method provides an equivalent effective-resolution improvement of about 5.76 bits under the target narrowband observation condition, thereby effectively enhancing the spectral purity and detectability of the error signal within the control loop.

It should be noted that the experimentally obtained improvement in effective resolution is still lower than the ideal theoretical value. This discrepancy can mainly be attributed to the following two factors. First, non-negligible low-frequency flicker noise (1/f noise) exists in the system. Such noise is concentrated mainly in the near-DC region and lies within the effective passband of the adopted digital decimation process; therefore, it cannot be completely suppressed by simple windowed averaging. Second, the overall system performance is also limited by the analog noise floor of the AD9364 receiver front end. Once the processing gain introduced by digital filtering reduces the noise level close to the physical noise limit of the analog front end, the signal-to-noise ratio no longer improves linearly with the averaging length, which ultimately causes the actual improvement in effective resolution to fall short of the theoretical prediction.

### 4.3. Comparison of Frequency Stability

**Figure 13:**
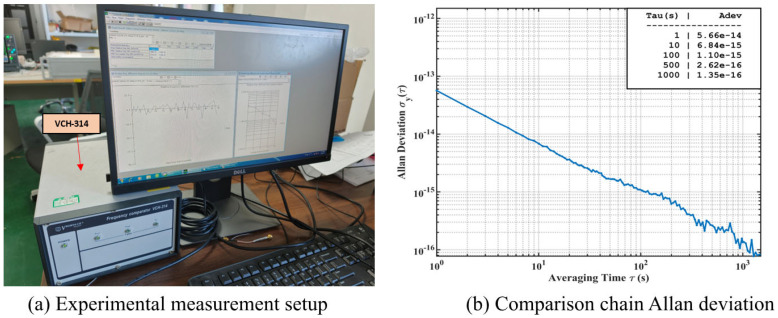
Frequency stability measurement setup and comparison chain evaluation. (**a**) Experimental measurement setup with VCH-314 high-precision frequency comparator. (**b**) Allan deviation obtained when both inputs of VCH-314 are connected to VCH1003M active hydrogen maser.

Using the proposed digital servo circuit, stable closed-loop locking of the PHM was achieved. To quantitatively evaluate the frequency stability performance of the developed digital servo system, a closed-loop comparison experiment was carried out using a VCH-314 high-precision frequency comparator, in which the output of the clock under test was continuously compared with that of a VCH1003M, an active hydrogen maser. The measurement setup is shown in Figure 13a. Since the VCH1003M provides substantially higher frequency stability over the observation interval, it can be used as a high-precision reference in this experiment. To further assess the residual noise contribution of the comparison chain, an additional test was performed by connecting both the reference and test inputs of the VCH-314 to the VCH1003M, and the corresponding Allan deviation is shown in Figure 13b. The result indicates that the residual stability level of the comparison chain remains below that of the PHM under test over the relevant averaging-time range. Therefore, the measured Allan deviation mainly reflects the frequency stability performance of the developed PHM servo system. The corresponding stability specifications of the reference source are listed in Table 2.

**Figure 14:**
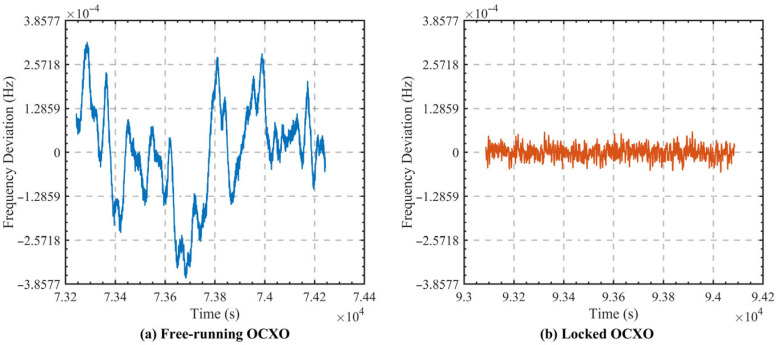
Relative frequency deviation under different operating conditions: (**a**) free-running; (**b**) closed-loop.

Figure 14 presents the comparison of frequency deviation under free-running and closed-loop locking conditions. In the free-running state, the system output exhibits pronounced random fluctuations, with a frequency deviation amplitude of approximately $1.2\times 10^{-3}\;\mathrm{Hz}$. After the digital closed-loop control is activated, the frequency deviation range becomes markedly narrower. Once the system reaches a stable locked state, the frequency fluctuation is compressed to within $1.6\times 10^{-5}\;\mathrm{Hz}$. These results demonstrate that the digital servo loop, built upon dual-frequency interrogation, error signal separation, and incremental PI control, can effectively correct the frequency offset of the local oscillator and achieve stable tracking around the center of the atomic transition.

**Figure 15:**
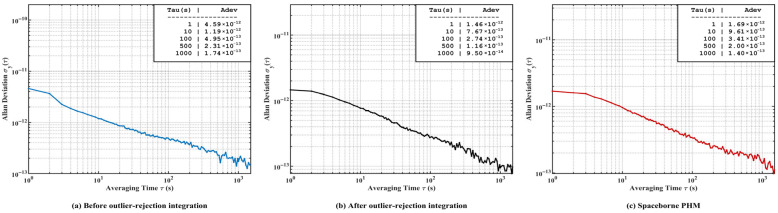
Comparison of Allan deviation before and after outlier rejection averaging decimation, together with the reference performance of a spaceborne PHM.

To further quantitatively evaluate the frequency stability of the system, an Allan deviation analysis was performed on the recorded frequency deviation sequence, and the results are shown in Figure 15. Figure 15a presents the Allan deviation curve of the system without applying the proposed outlier rejection and averaging decimation method. It can be observed that, under this condition, the system error signal is more evidently affected by random noise, and the short-term frequency stability is therefore limited, with a frequency stability of approximately $4.59\times 10^{-12}$ at τ=1 s.

On this basis, Figure 15b shows the Allan deviation results after introducing outlier rejection and averaging decimation. Compared with the untreated case, the short-term stability of the system is significantly improved, reaching $1.46\times 10^{-12}$ at τ=1 s. Meanwhile, the processed Allan deviation curve approximately follows a $\tau^{-1/2}$ trend over the test range, indicating that the system is mainly dominated by white frequency noise on this timescale. In addition, no obvious drift-dominated behavior appears at τ=1000 s, which demonstrates that the proposed outlier rejection and averaging decimation method can effectively suppress error signal noise and improve the mid- and short-term frequency stability of the system.

Figure 15c further compares the frequency stability performance of the proposed SDR-based digital servo system with that of the conventional spaceborne implementation, both evaluated on the same physics package. Over the averaging time range from 1 s to 1000 s, the proposed system exhibits frequency stability comparable to that of the conventional spaceborne implementation, reaching $1.46\times 10^{-12}$ at τ=1 s and $9.50\times 10^{-14}$ at τ=1000 s.

On this basis, the proposed system was further evaluated from the perspective of engineering implementation. According to the SWaP comparison results listed in Table 3, while achieving stable closed-loop locking of the PHM and the expected frequency stability performance, the proposed scheme reduces the overall volume by approximately 46.3%. The system mass is decreased from 6.21 kg to 2.5 kg, corresponding to a reduction of about 59.7%, and the steady-state power consumption is 34 W in the present implementation. Overall, the proposed SDR-based digital servo architecture satisfies the functional and performance requirements for PHM closed-loop locking, while providing a more compact and integrated implementation form. In particular, the reductions in system mass and volume are evident, indicating the engineering potential of the proposed architecture for future miniaturized PHM servo electronics.

## 5. Conclusions

This paper presents the design and implementation of an SDR- and FPGA-based digital servo architecture for PHM, targeting the demands of miniaturization and high integration in servo electronics. The proposed system employs the AD9364 as an integrated RF front end, while digital demodulation, cascaded decimation, envelope extraction, error signal construction, closed-loop control, and outlier rejection and averaging decimation are implemented in the FPGA, thereby realizing the digital integration of the conventional discrete analog servo chain. Experimental results demonstrate that the developed system can achieve stable closed-loop locking of the atomic transition spectrum, with a frequency stability of $1.46\times 10^{-12}$ at 1 s. In addition, the in-band noise power of the error signal is suppressed by approximately 34.68 dB, leading to a significant improvement in error signal observation accuracy as well as the mid- and short-term frequency stability of the system. Compared with conventional discrete implementations, the proposed architecture shows clear advantages in power consumption, volume, and integration level, thereby verifying the feasibility of the SDR-based digital servo approach for miniaturized passive hydrogen masers.

Future work will focus on further reducing the low-frequency noise floor and narrowing the gap between the experimentally obtained effective-resolution improvement and the ideal theoretical estimate. Possible directions include the adoption of a lower-noise RF front end, the introduction of more advanced noise-shaping or noise-suppressing strategies, and the use of more advanced digital filtering algorithms to improve weak error signal extraction.

## Figures and Tables

**Table 1:** Timing and Modulation Parameters of Interrogation System.

Parameter Name	Symbol	Design Value
Atomic interrogation duration	$T_{atom}$	4 s
Cavity interrogation duration	$T_{cav}$	0.4 s
Total interrogation cycle	$T_{cycle}$	4.4 s
Frequency Switching Interval	T	40 ms
Atomic Modulation Depth	$f_{osc}$	12.5 Hz
Cavity Frequency Modulation Depth	${\;f}_{cav}$	50 kHz

**Table 2:** Frequency stability of VCH1003M active hydrogen maser.

Time Domain	3 Hz Measuring Bandwidth
1 s	$\leq \;1.5\times 10^{-13}$
10 s	$\leq \;2.5\times 10^{-14}$
100 s	$\leq \;6.5\times 10^{-15}$
1000 s	$\leq \;2.0\times 10^{-15}$

**Table 3:** SWaP Parameter Comparison.

	Mass (kg)	Volume (cm^3^)	Power (W)
SDR-based PHM	2.5	3801.6	34
Spaceborne PHM	6.21	7075.6	36

## Data Availability

The original contributions presented in this study are included in the article. Further inquiries can be directed to the corresponding author.

## Abbreviations

The following abbreviations are used in this manuscript:

PHM	Passive Hydrogen Maser
SDR	Software Defined Radio
FPGA	Field-Programmable Gate Array
PI	Proportional–Integral
SWaP	Size, Weight, and Power
ENOB	Effective Number of Bits
ADC	Analog-to-Digital Converter
CAT	Cavity Varactor Diode
VLBI	Very Long Baseline Interferometry
ILA	Internal Logic Analyzer
DDS	Direct Digital Synthesizer
NCO	Numerically Controlled Oscillator
VCXO	Voltage-Controlled Crystal Oscillator
FSMC	Flexible Static Memory Controller
SPI	Serial Peripheral Interface
low-IF	Low-Intermediate-Frequency
PSD	Power Spectral Density
